# Th2-high asthma: a heterogeneous asthma population?

**DOI:** 10.1186/2045-7022-5-S2-O1

**Published:** 2015-03-23

**Authors:** Seys Sven, Hans Scheers, Gudrun Marijsse, Ellen Dilissen, Annelies Van Den Bergh, Pieter Goeminne, Paul Van den Brande, Jan Ceuppens, Lieven Dupont, Dominique Bullens

**Affiliations:** 1 University of Leuven, Clinical Immunology, Leuven, Belgium; 2 University of Leuven, Pneumology, Leuven, Belgium; 3 University Hospitals Leuven, Pneumology, Leuven, Belgium; 4 University of Leuven, Pediatric Immunology, Leuven, Belgium

## Background

Airway inflammation in asthma can be subdivided in Th2-high and Th2-low.

## Objective

To identify unique patient clusters with a specific airway cytokine expression profile in an unselected population with asthma.

## Methods

Induced sputum and clinical records were analysed from 208 asthmatics and 80 healthy individuals. Cytokine-high patients had cytokine mRNA levels above the 90^th^ percentile value in controls. Unsupervised hierarchical clustering was used to determine unique cytokine-based patient clusters.

## Results

Cubic Clustering Criterion, pseudo F and t^2^ statistics revealed a two- and a six-cluster model. The first cluster (n=23) was found in both models and consists of patients who present with an “IL-5-high and IL-17F-high” profile. Patients with an “IL-4- or IL-13-high” profile did not cluster in one single group. In the six-cluster model, the “IL-17F-low” group was divided into 5 separate clusters: “IL-5-high” profile (n=7), “IFN-γ-high” profile (n=15), “IL-6- and/or TNF-high” profile (n=15), “IL-22-high” profile (n=15) and those patients that were low for all preceding cytokines (n=130; cluster 6). “IL-17F- and IL-5-high” patients had significantly lower FEV_1_ and higher sputum neutrophils. Patients that were only “IL-4- or IL-13-high” (cluster 6) had highest F_E_NO levels and sputum eosinophils.

## Conclusion

Th2-high asthma can be subdivided in patients with “IL-5-high and IL-17F-high” asthma and those with “IL-4- or IL-13-high” asthma. The inflammatory pattern is different between both groups. The former group is characterized by mixed granulocytic airway inflammation whereas the latter group consists of patients with eosinophilic airway inflammation.

